# Physical mapping and InDel marker development for the restorer gene *Rf*_*2*_ in cytoplasmic male sterile CMS-D8 cotton

**DOI:** 10.1186/s12864-020-07342-y

**Published:** 2021-01-06

**Authors:** Juanjuan Feng, Xuexian Zhang, Meng Zhang, Liping Guo, Tingxiang Qi, Huini Tang, Haiyong Zhu, Hailin Wang, Xiuqin Qiao, Chaozhu Xing, Jianyong Wu

**Affiliations:** 1 State Key Laboratory of Cotton Biology, Institute of Cotton Research of Chinese Academy of Agricultural Sciences, 38 Huanghe Dadao, Anyang, 455000 Henan China; 2 Western Agricultural Research Center of Chinese Academy of Agricultural Sciences, 195 Ningbian East Road, Changji, 831100 Xinjiang China

**Keywords:** Cotton, CMS, *Rf*_*2*_, BSA, SNP, InDel

## Abstract

**Background:**

Cytoplasmic male sterile (CMS) with cytoplasm from *Gossypium Trilobum* (D8) fails to produce functional pollen. It is useful for commercial hybrid cotton seed production. The restore line of CMS-D8 containing *Rf*_*2*_ gene can restore the fertility of the corresponding sterile line. This study combined the whole genome resequencing bulked segregant analysis (BSA) with high-throughput SNP genotyping to accelerate the physical mapping of *Rf*_*2*_ locus in CMS-D8 cotton.

**Methods:**

The fertility of backcross population ((sterile line×restorer line)×maintainer line) comprising of 1623 individuals was investigated in the field. The fertile pool (100 plants with fertile phenotypes, F-pool) and the sterile pool (100 plants with sterile phenotypes, S-pool) were constructed for BSA resequencing. The selection of 24 single nucleotide polymorphisms (SNP) through high-throughput genotyping and the development insertion and deletion (InDel) markers were conducted to narrow down the candidate interval. The pentapeptide repeat (PPR) family genes and upregulated genes in restore line in the candidate interval were analysed by qRT-PCR.

**Results:**

The fertility investigation results showed that fertile and sterile separation ratio was consistent with 1:1. BSA resequencing technology, high-throughput SNP genotyping, and InDel markers were used to identify *Rf*_*2*_ locus on candidate interval of 1.48 Mb on chromosome D05. Furthermore, it was quantified in this experiment that InDel markers co-segregated with *Rf*_*2*_ enhanced the selection of the restorer line. The qRT-PCR analysis revealed PPR family gene *Gh_D05G3391* located in candidate interval had significantly lower expression than sterile and maintainer lines. In addition, utilization of anther RNA-Seq data of CMS-D8 identified that the expression level of *Gh_D05G3374* encoding NB-ARC domain-containing disease resistance protein in restorer lines was significantly higher than that in sterile and maintainer lines.

**Conclusions:**

This study not only enabled us to precisely locate the restore gene *Rf*_*2*_ but also evaluated the utilization of InDel markers for marker assisted selection in the CMS-D8 *Rf*_*2*_ cotton breeding line. The results of this study provide an important foundation for further studies on the mapping and cloning of restorer genes.

**Supplementary Information:**

The online version contains supplementary material available at 10.1186/s12864-020-07342-y.

## Background

The cytoplasmic male sterility (CMS) system plays an important role in utilization of crop heterosis. CMS is a maternally inherited trait, that includes degenerate anthers, aborted pollen with carpelloid and petaloid stamens [1]. Current research has determined that the CMS phenotype is caused by mutations in the mitochondrial genome linked genes and reserved by fertility restorer genes in the nuclear genome [2–4]. The CMS system avoids the removal of anthers, thereby enabling the generation of dramatically superior F_1_ progenies through hybrid technology. These offsprings display significant advantages over their parents and existing popular cultivars in terms of yield, stress tolerance, adaptability, etc. [5]. The CMS phenomenon exists in more than 150 plants and is also used for hybrid breeding of crops, such as maize [6, 7], rice [8, 9], pepper [10] and sorghum [11].

Cotton (*Gossypium hirsutum L.*) is a vital source of fibre, oil, and the most important economic crop for the textile industry in the world. In cotton, the CMS system is an ideal way to improve hybrid yields [12], *Harknessii* (D_2–2_) cytoplasmic male sterile (CMS-D2) lines [13, 14], *Trilobum* (D8) cytoplasmic male sterile (CMS-D8) lines [15], and upland cotton cytoplasmic male sterile (104-7A, Xiangyuan A, Jin A) have been established and utilized [16]. Normally, different CMS lines could be recovered by different restorer genes. In cotton, the restorer gene *Rf*_*1*_ of CMS-D2 could restore the fertility of CMS-D2 and CMS-D8 sterile lines, while fertility of CMS-D8 sterile lines could only be restore with *Rf*_*2*_ [17]. Furthermore, the *Rf*_*1*_ gene functions in sporophytes, whereas the *Rf*_2_ gene has a gametophytic restoration system. Previous studies revealed that *Rf*_*1*_ gene loci and *Rf*_*2*_ gene loci are not allelic, but these genes are tightly linked at a genetic distance of 0.93 cM on chromosome D05. The mapping and identification of the molecular markers linked with the *Rf*_*1*_ restorer gene in cotton has already been progressed [18–23]. However, there are few researches about the *Rf*_*2*_, compared with *Rf*_*1*_.

With the increase in crop functional genome research, *Rf* genes have been successfully cloned in maize (*Rf*_*2*_) [24], petunia (*Rf****-****PPR592*) [25], radish (*Rfo*) [26, 27], rice (*Rf1a*, *Rf1b, Rf2*) [28–31], sorghum (*Rf1*) [32], and sugar beet (*Rf1*) [33]. Most of these genes encode PPR proteins, but *Rf*_*2*_ in maize CMS-T, *Rf17* in rice CMS-CW and *Rf2* in rice CMS-LD encode aldehyde dehydrogenase, 178-amino-acid mitochondrial sorting protein and mitochondrial glycine-rich protein, respectively [24, 34, 35]. At present, the major bottleneck of cotton CMS breeding system is a narrow source of restorer genes and lack of excellent restorer lines compatible with a given sterile line. Unfortunately, no restorer gene has been cloned in cotton. Therefore, fine mapping and isolation of the restorer gene *Rf*_*2*_ in upland cotton are highly needed for efficient breeding. Interestingly, bulked segregant analysis (BSA) make it possible to quickly locate molecular markers closely linked to the target gene by analysing the differences between SNPs and InDels in segregating population pools [36]. This method has already been used in gene mapping of *Arabidopsis thaliana* [37], rice [38–40] maize [41] and tomato [42]. The SNP and the InDel are the most abundant type of DNA sequence polymorphisms, found within the genomic sequence of each species [43, 44], and used in QTL analysis. These markers have widely been used in cultivar identification, construction of genetic maps, genetic diversity, map-based cloning, the detection of genotype/phenotype associations, and marker-assisted breeding (MAS) [45–47]. In recent years, the release of the upland cotton genomic sequence [48–50] and the rapid development of sequencing technology have enhanced the detection and application of SNP and InDel. Furthermore, the application of high-throughput genotyping methods makes SNP highly attractive genetic markers [51, 52].

The objectives of this study were to physically map restorer gene *Rf*_*2*_ and to develop InDel markers co-segregated with *Rf*_*2*_. A 1.88 Mb candidate interval was obtained by combining BSA with high-throughput SNP genotyping using a BC_1_F_1_ segregation population. Based on the InDel variation in the 1.88 Mb interval, the InDel markers were developed and used to narrow down a 1.48 Mb candidate interval. The PPR family genes and the genes selected by transcriptome data in candidate region were analysed by qRT-PCR. The InDel markers co-segregated with *Rf*_*2*_ will be useful to trace *Rf*_*2*_ breeding restorer lines in cotton.

## Results

### Anther observation and BC_1_F_1_ fertility analysis

The anthers of fertile plants had a large amount of pollen, while the sterile plants had no pollen, and their anthers did not crack. Overall, a total of 1623 BC_1_F_1_ plants were classified as 850 fertile and 773 sterile plants, and the ratio of the number of fertile plants (850) to the number of sterile plants (773) fit a 1:1 segregation (χ2= 3.6531 < χ2_(0.05,1)_ = 3.84), confirming that fertility restoration is conditioned by one dominant restorer gene, *Rf*_*2*_. This result is consistent with the results of Zhang et al. [53].

### Whole genome resequencing data analysis and evaluation

The two parent lines (maintainer line B and restorer line R), F-pool, and S-pool of the BC_1_F_1_ segregation population were sequenced. The Illumina platform was selected to construct the paired-end (PE) library, and the PE fragment was between 300 and 500 bp; 1,251,289,091 reads were obtained (Table 1). The reads from samples were aligned to the reference genome using BWA software, with > 82.57% normal efficiency. For the sequencing results, the average Q30 was 94.95%, and the average GC content was 37.22%. A total of 177,874,504 reads were obtained for the R restorer line, with a Q30 value of 94.69%, and average GC content of 37.77%. On the other hand, 174,907,610 reads were obtained for the B maintainer line, with a Q30 value of 94.29%, and an average GC content of 36.69%. Finally, 465,660,282 and 432,846,695 reads were obtained for the filial BC_1_F_1_ generation (fertile and sterile) with Q30 values of 94.83 and 95.97%, and average GC content of 36.93 and 37.22%, respectively (Table 1).

**Table 1:** Results of high-throughput resequencing data mining

Sample	Reads	Bases	GC(%)	Q20(%)	Q30(%)
B	174,907,610	52,032,498,409	36.69	98.43	94.29
R	177,874,504	52,866,884,655	37.77	98.57	94.69
fertility-bulk	465,660,282	138,509,668,729	37.50	98.53	94.83
sterility-bulk	432,846,695	128,892,417,044	36.93	98.90	95.97
Mean	312,822,273	93,075,367,209	37.22	98.61	94.95
Sum	1,251,289,091	372,301,468,837	–	–	–

### BSA combining SNP-index and G’ values

The average sequencing depth of the parent lines and the offspring pools was 30.92×. Of these, the R restorer line has a sequencing depth of 16.62×. The B maintainer line sequencing depth was 16.03×, whereas the sequencing depth of the filial BC_1_F_1_ generation was 47.77× + 43.26× (Table 2).

**Table 2:** Sequencing coverage and depth data

Sample	Coverage(%)	Mean Depth
B	82.94	16.03
R	79.95	16.62
fertility-bulk	83.44	47.77
sterility-bulk	83.93	43.26
Mean	82.57	30.92

**Fig. 1:**
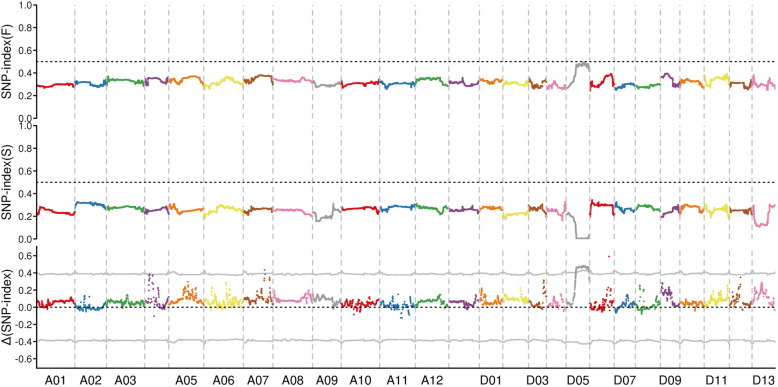
SNP-index algorithm to map the *Rf*_*2*_ gene. The coloured point represents the calculated SNP-index (or ΔSNP-index) value. The top graph illustrates the distribution of the SNP-index values in the F mixed pool; the middle graph shows the distribution of the SNP-index values in the S mixed pool; the bottom graph shows the distribution of the ΔSNP-index values, and the grey line represents the theoretical threshold line

**Fig. 2:**
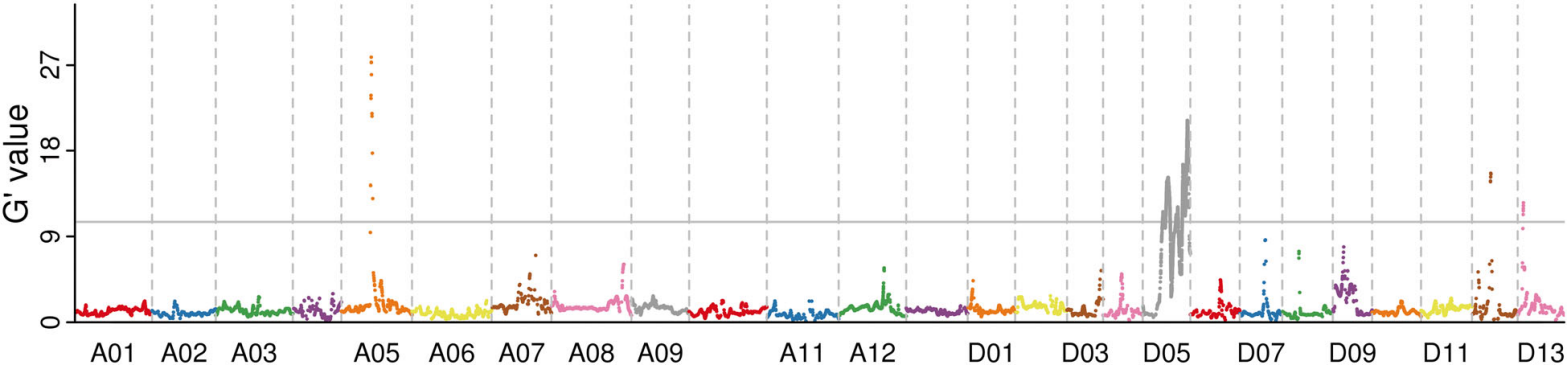
G’ algorithm to map the *Rf*_*2*_ gene. The distribution of G’ values on the chromosome. Note: The abscissa is the chromosome name. The colour point represents the G’ values of each SNP locus. The grey line represents the threshold of significant association. The higher the G’ value, better is the correlation effect

These reads were mapped onto the reference genome of *Gossypium hirsutum* (Tm-1, http://mascotton.njau.edu.cn/info/1054/1118.htm). A total of 798,286 SNPs was obtained from the two mixed pools, and 72,108 small InDel were obtained from the mixed pools. We used two different methods to map the *Rf*_*2*_ locus responsible for restoring fertility. As shown in Figs. 1 and 2, only one locus was identified, and both the SNP-index and G’ value association algorithms mapped this locus to chromosome D05. More specifically, this locus was located in the region of 25.61 Mb–59.94 Mb (34.33 Mb) using the SNP-index and G’ value method.

### Fine mapping of the *Rf*_2_ gene

**Fig. 3:**
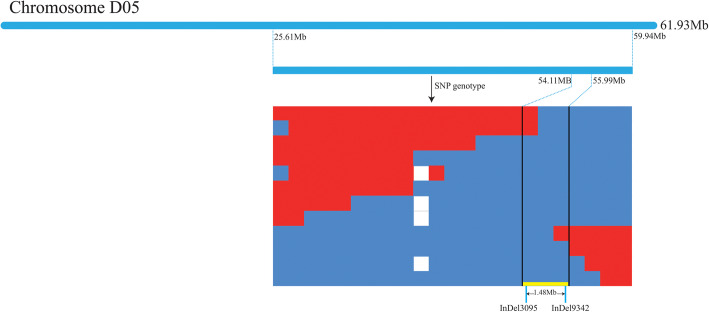
Molecular mapping of the *Rf*_*2*_ gene using the SNP/InDel combinational approach. White indicates a lack of sample, red indicates that the SNP site was exchanged, and blue indicates that the genotype and phenotype were consistent

**Fig. 4:**
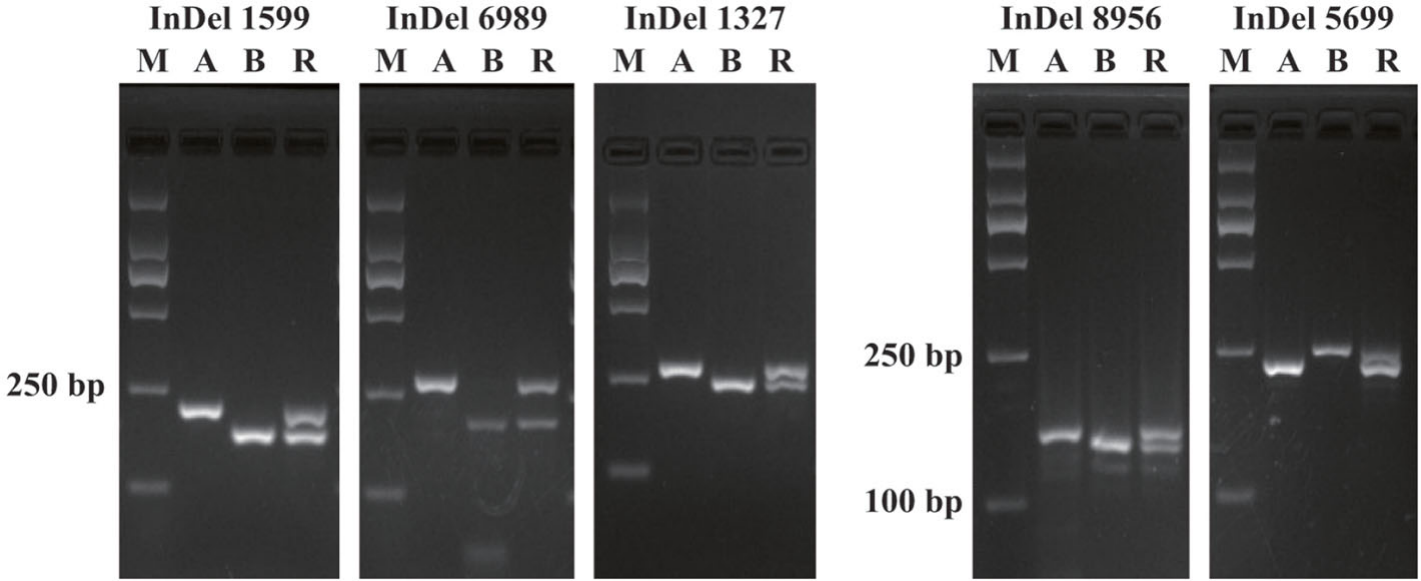
The InDel markers co-separating with *Rf*_*2*_, *A* sterile line, *B* maintainer line, *R* restorer line, the full gel pictures are supplied in Fig. S1 and Fig. S2

It was difficult to determine the candidate gene of *Rf*_*2*_, since the candidate range of 34.33 Mb contains a large amount of genetic information. Thus, it was necessary to fine map *Rf*_*2*_. We developed 24 SNP markers, and 23 valid SNP markers in this region were used for genotyping an additional 1423 individuals by high-throughput SNP genotyping. We found 6 recombinant plants in the BC_1_F_1_ population. The position of the *Rf*_*2*_ locus was narrowed down and was located between SNP563981 and SNP597385, a 1.88 Mb region (Supplementary Table S2, the information of the SNP site). Next, we developed InDel markers on the correlated region, and InDel marker analyses revealed that 16 InDel markers were polymorphic. These InDel markers narrowed down the candidate interval to 1.48 Mb for existing recombinants at InDel marker sites. Finally, 10 InDel markers were co-segregated with *Rf*_*2*_ (Figs. 3 and 4, Supplementary Table S3, InDel primers).

### Marker-assisted breeding of restorer lines and CMS-D8 hybrid identification

Subsequently, 500 plants were randomly selected from the BC_4_F_1_ population of CMS-D8, and the InDel 1327 marker was used for genotype analysis. The BC_4_F_1_ population was typed by visual fertility investigations. The PCR products were analysed by agarose gel electrophoresis, and the results of agarose gel electrophoresis showed two different banding patterns. A single small PCR product was considered homozygous and lacked the restorer gene allele [S (*rf*_*2*_*rf*_*2*_)], indicating sterile plants, whereas two fragments were considered heterozygous at the restorer gene locus [S (*Rf*_*2*_*rf*_*2*_)], indicating fertile plants. Furthermore, the segregation ratio followed a 1 (*Rf*_*2*_*rf*_*2*_):1 (*rf*_*2*_*rf*_*2*_) (254 *Rf*_*2*_*rf*_*2*_: 246 *rf*_*2*_*rf*_*2*_, χ^2^_0.05_ = 0.1667 < 3.841), and the results were consistent with those of the fertility survey.

**Fig. 5:**
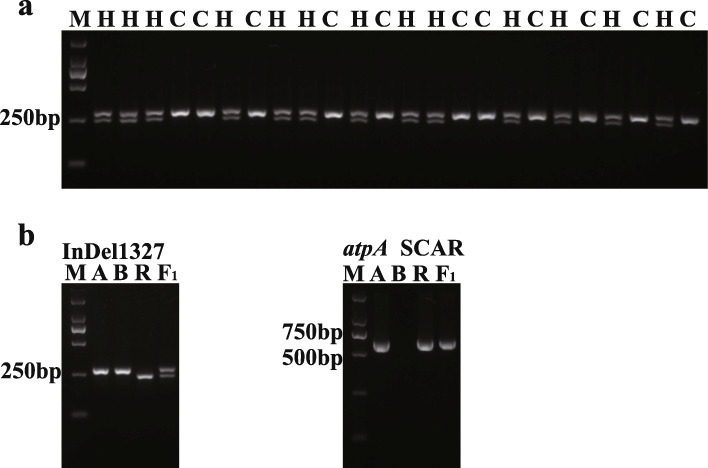
**a** BC_4_F_1_ plants were screened with InDel 1327, *M* marker, *H Rf*_*2*_ heterozygous plants, *C* plants lacking the restorer gene *Rf*_*2*_, the full gel picture is supplied in Fig. S3. **b** Molecular identification of the CMS system hybrids and cotton varieties with InDel 1327 and *atpA* SCAR markers. *M* DL2000 DNA marker; *A* sterile line, *B* maintainer line, *R* restorer line, *F*_*1*_ A line ×R line, the full gel pictures are supplied in Fig. S4 and Fig. S5

The plants were scanned with an *atpA* SCAR marker [2] and InDel 1327 markers, as the hybrids of the CMS-D8 system have sterile cytoplasm and *Rf*_*2*_ heterozygous sites. The InDel 1327 primer amplification product produced two bands, and the *atpA* SCAR marker amplification product produced a band with a size of 611 bp (Fig. 5 b). Therefore, the CMS-D8 hybrids with heterozygous restorer gene sites and sterile cytoplasm were differentiated by the genotyping of restorer genes and the identification of cytoplasm type.

### Candidate gene selection and expression pattern analysis

**Fig. 6:**
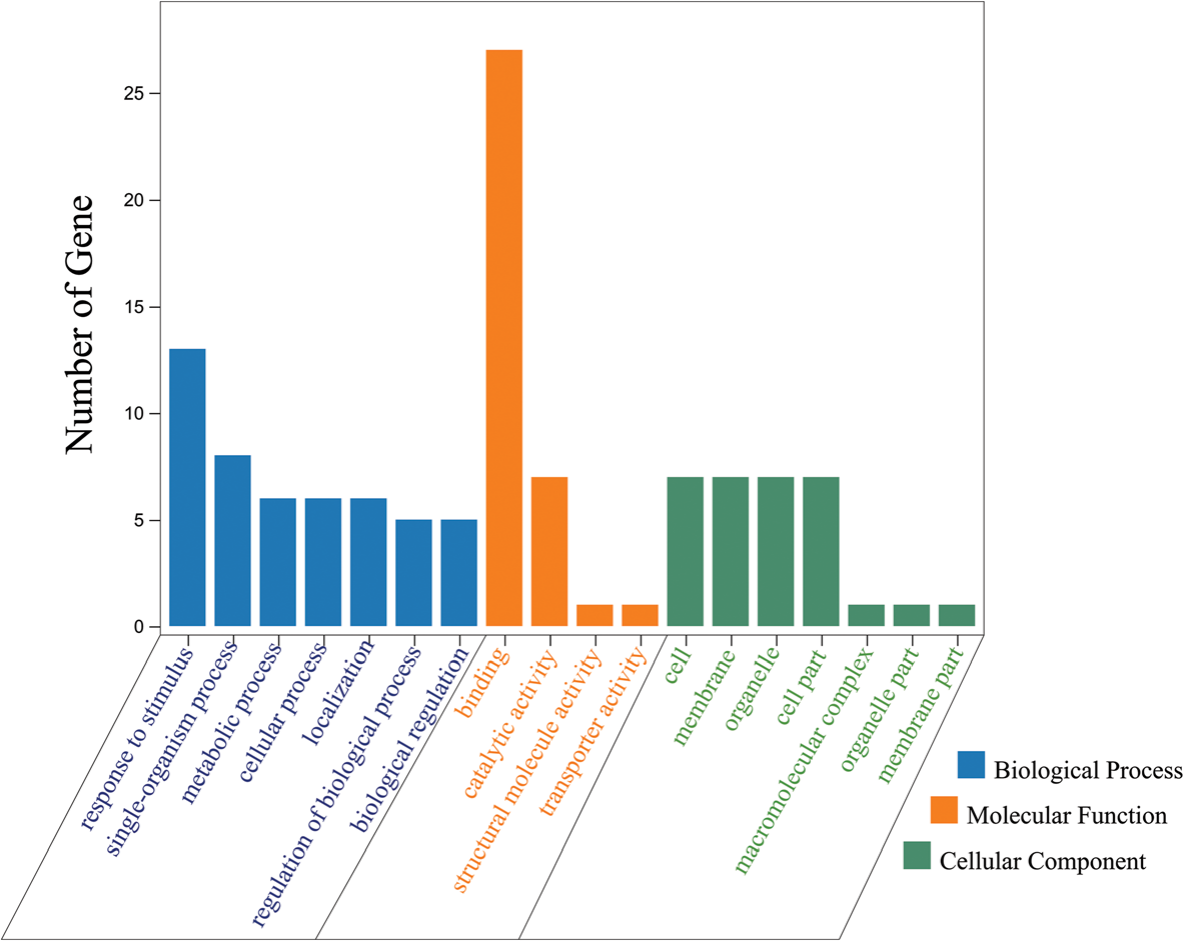
Gene Ontology (GO) analysis of 67 genes in the candidate interval

**Fig. 7:**
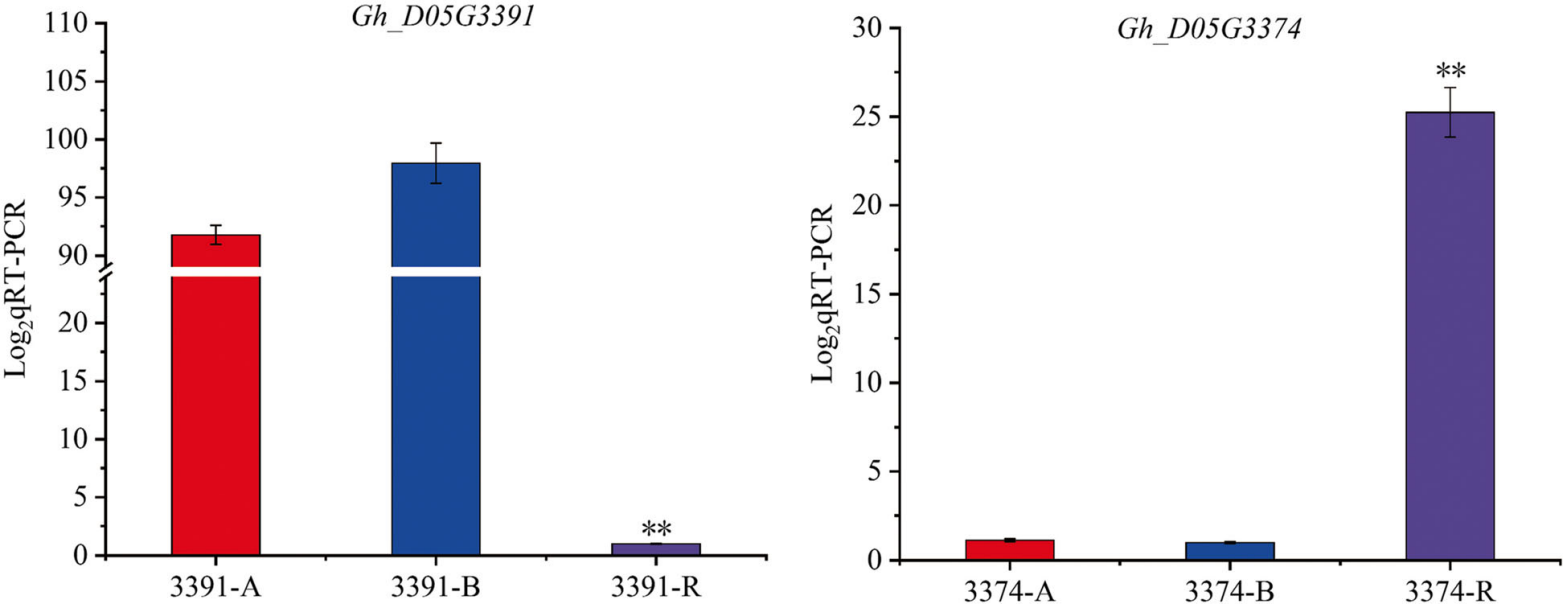
Expression patterns of *D05G3391* and *D05G3374*. (**, *P* < 0.01) The asterisks indicate that the difference in gene expression in the A, B and R lines was highly significant

To determine candidate genes, we adopted a method that combined the 67 genes in the interval with the functional annotation of *Arabidopsis* orthologues and transcriptome data [54]. The 67 genes were subjected to Gene Ontology (GO) analysis. The GO analysis indicated that most of the genes are involved in binding (Fig. 6). According to the successfully isolated restorer genes of other crops belonging to the PPR family, we used qRT-PCR to analyse PPR family genes in candidate interval. Interestingly, the candidate region of the *Rf*_*2*_ locus was found to contain 8 PPR genes (*Gh_D05G3356, Gh_D05G3357, Gh_D05G3359, Gh_D05G3378, Gh_D05G3389, Gh_D05G3391, Gh_D05G3392, Gh_D05G3380*) in the region of 1.48 Mb. The relative expression levels of the eight PPR family genes in the restorer line were not significantly higher than those of the maintainer and the sterile lines. However, the relative expression of the *Gh_D05G3391* gene in the restorer line was significantly lower than that in the sterile and maintainer lines (Fig. 7).

Furthermore, the *Gh_D05G3374*, *Gh_D05G3407* and *Gh_D05G3417* genes were chosen based on FDR< 0.05 and |log_2_FC|>= 1 by the RNA sequence data (Supplementary Table S4). Since, the expression level in the restorer line was significantly higher than that in the sterile and maintainer lines. The qRT-PCR results showed that the *Gh_D05G3417* gene in the restorer line was significantly higher than that in the sterile and maintainer lines (Fig. 7). Finally, two genes (*Gh_D05G3391* and *Gh_D05G3374*) were selected as possible candidate genes.

## Discussion

CMS is a common phenomenon that occurs in flowering plants due to interactions between the mitochondrial genome and the nuclear genome [55]. CMS systems have been proven to be a proficient tool in hybrid seed production. Considering the importance of the CMS and restoration systems, numerous molecular mapping studies have been performed on restorer genes in crops, and *Rf* genes have already been isolated in other crops [24–33]. With CMS systems in cotton, fertility can be restored by restoring the genes *Rf*_*1*_ or *Rf*_*2*_. However, these two genes have not yet been identified and cloned. With the availability of upland cotton whole genome sequencing [48–50] and cotton mitochondrial genome sequencing [56], breakthroughs in the study of cotton CMS and restoration of fertility mechanisms can be realized in recent years.

### Molecular marker discovery and fine mapping of the fertility restoring gene of CMS cotton

Some researchers have recently studied cotton CMS systems for molecular marker development and fine mapping of fertility restoration genes. For instance, Liu et al. [18] identified 2 RAPD and 3 SSR markers closed linked with *Rf*_*1*_. Feng et al. [19] developed 4 STS markers associated with *Rf*_*1*_. Yin et al. [20] constructed a BAC library of CMS-D2 restorer lines and reported that *Rf*_1_ was located 100 kb between two BAC clone overlapping regions. Yang et al. [57] identified 6 EST-SSR markers (NAU2650, NAU2924, NAU3205, NAU3652, NAU3938, and NAU4040) with a genetic distance of 0.327 cM linked to *Rf*_*1*_ of CMS-D2. Wu et al. [21] screened 13 molecular markers closely linked to *Rf*_*1*_ and located *Rf*_*1*_ between the SSR markers BNL3535 and NAU3652, with genetic distances of 0.049 cM and 0.078, respectively. Recently, they have reported co-segregated InDel markers such as InDel-1891, InDel-3434, InDel-7525, InDel-9356 and InDel-R [22, 58]. Zhao et al. [23] used super-BSA and successfully mapped *Rf*_*1*_ to 1.35 Mb region of chromosome D05. Previous studies have shown that *Rf*_*1*_ and *Rf*_*2*_ are tightly linked at a genetic distance of 0.93 cM on chromosome D05 [17]. The findings of Wang et al. [59] revealed that CIR179–250 was strictly linked with both *Rf*_*1*_ and *Rf*_*2*_, which was located on chromosome D05(19th chromosome). The present study on the *Rf*_*2*_ gene identified the location of the chromosome D05 base sequence as 54.3–55.78 Mb. Furthermore, the present study developed 10 InDel markers in the correlated region. These markers laid the foundation for locating and fine mapping *Rf*_*2*_ in CMS-D8 cotton.

### Mapping *Rf*_*2*_ using an efficient strategy

Traditional map-based cloning is an efficient approach to isolate genes/QTLs responsible for desired agronomic traits [60–62]. Usually, a genetic map of F_2_, double haploid (DH) or recombinant inbred line (RIL) populations based on hundreds of SSR or InDel markers is used to make a primary map. Then a near-isogenic line (NIL) is developed which based on MAS to narrow down the region of interest to a sufficient size to screen for a few candidate genes. Unfortunately, this workflow requires relatively more labour and time [63]. Compared with genetic mapping, the next generation sequencing (NGS) is a faster and reliable method for mapping [64]. Nevertheless, one mixed pool typically contains approximately 20–100 individuals and generally maps the target region at a Mb-level interval [65–67]. Because of insufficient meiotic recombination events, researchers still have to perform fine mapping or use omics methods such as RNA-seq to further screen the candidate genes [68, 69].

High-throughput SNP genotyping is one of the dimorphic methods in which genotypes are confirmed by direct sequencing [70]. It has been successfully used to genotype interesting traits in plants [71, 72]. In this regard, Yang et al. [73] developed 1536 SNP markers to measure genetic diversity by a high-throughput SNP genotyping method.

In this study, SNP-index and InDel-index analyses were used to first position the *Rf*_*2*_ gene within a 34.33 Mb region. Later on, twenty-three SNP sites selected in this region helped to narrow the *Rf*_*2*_ gene to a 1.88 Mb region. We developed InDel markers based on InDel variations and used these markers to locate the *Rf*_*2*_ gene in a 1.48 Mb region. We thus put forward an approach that could rapidly fine map gene loci using only a large BC_1_F_1_ segregation population, especially for those traits governed by single nuclear-encoded genes. This can be achieved by developing a large segregation population, mapping by sequencing analysis, and high-throughput SNP genotyping in a short time. Moreover, rapid and accurate identification of phenotype can be performed with progeny tests for desired objectives. Our study results suggested that BSA-seq combined with SNP genotyping can accelerate the mapping of loci controlling quality traits.

### Utilization of InDel markers for MAS

Development of DNA markers linked to agronomically important traits and their use for MAS plays the role in promoting variety [74]. And various types of molecular markers closely linked to cotton restorer genes have been developed [19, 21, 57], but these markers are difficult to use for molecular marker-assisted breeding because of the complex experimental processes or low sensitivity of the markers [22]. Very recently, PCR based InDel have become a popular gel based genotyping solution, since InDel has the advantages of co-dominant, inexpensive, and highly polymorphic nature [44, 75]. In this study, InDel markers co-segregated with restorer genes tracked *Rf*_*2*_ for molecular marker-assisted breeding. InDel markers developed on the region showed a higher identification rate of the *Rf*_*2*_ phenotype than previously developed markers, when applied to the breeding improvement of restorer lines.

### Characteristics of the potential candidate gene *Rf*_*2*_

Currently, *Rf* genes have been successfully isolated from different crop species [76]. Most of these restorer genes belong to the PPR gene family. PPR-type fertility restorer genes have been cloned for petunia [25], Ogura and Kosena cytoplasm in *Raphanus sativus* [26, 27, 77], BoroII CMS in *Oryza sativa* [78], A1 cytoplasm in *Sorghum bicolor* [32], Honglian CMS in rice [28, 29], and nap CMS in *Brassica napus* [79]. In this study, we explored the expression patterns of 8 PPR genes of the CMS-D8 system in the candidate interval, and the expression level of most genes in the restorer line was not significantly different from that of the sterile and the maintainer lines. Interestingly, *D05G3391*, a PPR family gene had significantly lower expression in restorer line than in the sterile and maintainer lines.

However, non-PPR restorer genes also exist in other crops, *Rf2* in the maize CMS-T system encodes aldehyde dehydrogenase [24], *Rf17*of CMS-CW system and *Rf2* of CMS-LD system in rice encode 178-amino-acid mitochondrial sorting protein and mitochondrial glycine-rich protein [34, 35]. Likewise, transcriptome data (unpublished data) was used to select upregulated genes of restorer line and then analysed by qRT-PCR. The relative expression of *D05G3374*, an NB-ARC disease-resistant protein gene, was significantly higher in restorer line than in sterile line and maintainer line. Based on the results of qRT-PCR, the candidate genes were not determined with the desired results in this study. The reason may be that *Rf*_*2*_ is derived from the nuclear gene of *G*. *trilobum* [15] and might not be available in the reference genome of *G. hirsutum*. Therefore, cloning of fertility restorer genes in cotton CMS systems still needs further investigation.

## Conclusions

In our study, the BC_1_F_1_ population was chosen as a genetic population to map *Rf*_*2*_ of CMS-D8. Integration of BSA, high-throughput SNP genotyping, and InDel markers identified 1.48 Mb candidate interval on chromosome D05. The InDel markers co-segregated with *Rf*_*2*_ can be used to trace *Rf*_*2*_ for molecular marker-assisted breeding of restorer lines or hybrids. The qRT-PCR analysis identified *Gh_D05G3391* and *Gh_D05G3374* genes as a putative candidate in this interval. The InDel markers co-segregated with *Rf*_*2*_ can be used not only to trace *Rf*_*2*_ for molecular marker-assisted CMS breeding but also cornerstone in fine mapping and cloning of restorer genes in cotton.

## Methods

### Materials and sample collection

The CMS-D8 system, a sterile line (A), maintainer line (B) and restorer line (R) were provided by the Institute of Cotton Research (ICR), Chinese Academy of Agricultural Science, Anyang, Henan, China. A BC_1_F_1_ ((A line ×R line) ×B line) segregation population was constructed, and all materials were grown at the Cotton Research Farm at the ICR. Fresh leaves were obtained from the parent lines and BC_1_F_1_ population. Anthers from buds1–2 mm, 3 mm, and 4 mm in length were collected and combined from 100 plants. All harvested samples were snap-frozen in liquid nitrogen and stored at − 80 °C before use.

### Fertility segregation analysis

During anthesis, visual fertility surveys were conducted for 1623 individuals of the BC_1_F_1_ population of CMS-D8 under field trial conditions, three times per plant. The presence of pollen in a plant indicated fertility and was determined by squeezing the anthers between the fingers because the male sterility of CMS-D8 occurs during meiosis, the *S (rf)* gametes are sterile, and *S (rf*_*2*_*rf*_*2*_*)* produces no pollen. So, the observed values of fertile and sterile plants were obtained, the fertility trait segregation ratio compatibility test was carried out using Excel software, and the Chi-square value was obtained to determine the genetic model of *Rf*_*2*_.

### DNA extraction, library construction and Illumina sequencing

DNA was extracted by the CTAB method [80], and the quality of DNA was assessed by 1.2% agarose gel electrophoresis. The purity of DNA was examined using an Agilent Technologies 2100 Bioanalyzer. The DNA concentration was estimated using a Qubit® DNA Assay Kit in a Qubit® 2.0 Fluorometer (Life Technologies, CA, USA). Equal amounts of DNA (1.5 μg/sample) from 100 BC_1_F_1_ plants with fertile phenotypes were mixed to form the fertile sample (F-pool), and those from another 100 plants with sterile phenotypes were mixed to form the sterile sample (S-pool). Sequencing libraries were generated using the VAHTS™ Universal DNA Library Prep Kit for Illumina® V3 (Vazyme Biotech) according to the manufacturer’s recommendations. Briefly, the DNA samples were fragmented by sonication to a size of 300–500 bp. Then, the DNA fragments were end-polished, A-tailed, and ligated with the full-length adapter for Illumina sequencing by PCR amplification. Consequently, the PCR products were purified (VAHTS™ DNA Clean Beads (Vazyme #N411)), and libraries were analysed for size distribution by an Agilent 2100 Bioanalyzer and quantified by real-time PCR. The libraries constructed above were sequenced by the Illumina HiSeq platform, and 150 bp paired-end reads were generated with an insert size of approximately 350 bp. The sequence raw reads were submitted to the SRA database of NCBI under the Bioproject PRJNA685585.

### Data analysis, data filtering, and alignment

The Fast x-toolkit (v 0.0.14–1) was used to filter out the low-quality reads such as reads with ≥10% unidentified nucleotides (N), reads with > 50% bases having phred quality < 5, reads with > 10 nt aligned to the adapter, allowing ≤10% mismatches, and putative PCR duplicates generated by PCR amplification in the library construction process (read 1 and read 2 of two paired-end reads were completely identical). The released genome of *Gossypium hirsutum* was downloaded from the Cotton Research Institute (CRI) of Nanjing Agricultural University of China (http://mascotton.njau.edu.cn/Data.htm, v1.1) and used as a reference genome [49]. For mapping to the reference genome, BWA (Burrows-Wheeler Aligner) [81] was used to align the clean reads of each sample against the reference genome (settings: mem -t 4 -k 32 –M -R). Alignment files were converted to BAM files using SAMtools software [82] (settings: –bS –t). In addition, potential PCR duplications were removed using the SAMtools command “rmdup”. If multiple read pairs had identical external coordinates, only the pair with the highest mapping quality was retained.

### SNP/InDel detection and annotation

Variant calling was performed for all samples by using the unified genotyper function in GATK [83] software. SNP was used as the variant filtration parameter in GATK (settings: --filterExpression “QD < 4.0 || FS > 60.0 || MQ < 40.0”, −G_filter “GQ < 20”, −-cluster WindowSize 4). InDel was filtered by Variant Filtration parameter (settings: --filter Expression “QD < 4.0 || FS > 200.0||Read PosRankSum < -20.0 || Inbreeding Coeff < -0.8”). ANNOVAR [84], an efficient software tool, was used to annotate the SNPs and InDels based on the GFF3 files for the reference genome.

### Determination of a candidate interval using SNP index and G’ values

To obtain a highly accurate SNP set, a range of filters were also employed [85]. The homozygous SNPs between two parents were extracted from the VCF files for SNPs. The read depth information for homozygous SNPs in the above offspring pools was obtained to calculate the SNP index [36]. The SNP index method was used for the analysis, and the SNP-index dot matched curve was obtained by regression fitting as described by Abe et al. [40]. The G’ values are used for noise reduction while also addressing linkage disequilibrium (LD) between SNPs [86]. To rule out the effects of unreliable markers, we screened markers based on the SNP index using the following conditions: 1) the sequencing depth of both parents was greater than 8; 2) both pools had a sequencing depth greater than 10; 3) the SNP index values of the two pools were not greater than 0.8 or less than 0.2 at the same time; and 4) the SNP index value difference was greater than 0.8. Sliding window methods were used to present the SNP index of the whole genome. Usually, we used a window size of 2 Mb as the default settings, and used QTL-seqr software to calculate Δ (SNPs-index) and G’, based on the markers that were scanned [87].

### Fine mapping of *Rf*_*2*_ using high-throughput SNP genotyping and InDel makers

For the analysis, DNA was isolated from the two parental lines and the BC_1_F_1_ population. The informative molecular markers were used for genotyping each plant of the BC_1_F_1_ population, various recombinants in the target region were identified, and the linkage relationship between markers and the *Rf*_2_ locus was analysed for gene mapping. According to the results of sequencing mutation detection and BSA of sequencing candidate intervals, one SNP was selected for approximately every 1.5 Mb of physical distance. The selected SNPs met the requirement of having a variation index close to 0.5 in the F-pool and 0 in the S-pool. A subset of 24 selected SNPs was used for genotyping by the Illumina HiSeq PE150 sequence. Individual BC_1_F_1_ population (except 200 plants that formed pools) plants were genotyped using high-throughput SNP genotyping. Based on the SNP locus exchange of individual plants, we narrowed down the range in which the target gene was located.

The InDel markers were developed based on the insertion deletion mutation within the selection interval. Primers were designed using Oligo7 software [88] and synthesized commercially (TSINGKE Biological Technology, Zhengzhou, China). The PCR system consisted of 20 μL of PCR mixture that contained 1× reaction buffer, 2.0 mM MgCl_2_, 0.2 mM dNTPs, 0.5 mM each primer, 1 U Taq DNA polymerase (Takara, Japan), and 50 ng of DNA template. The PCR amplification conditions were as follows: 35 cycles of denaturation at 94 °C for 30 s, annealing at 56 °C–58 °C for 30 s, and extension at 72 °C for 30 s. Then, the reaction was held at 4 °C. The PCR products were visualized by 3.5% agarose gel electrophoresis. Based on the difference between the genotypes as assessed using polymorphic markers, recombinants were identified in the BC_1_F_1_ population and used to fine map *Rf*_*2*_.

### Marker-assisted breeding of restorer lines

The utility of the InDel markers for marker-assisted selection was determined in a segregating population. First, the restorer line [*N (Rf*_*2*_*Rf*_*2*_*)*] of CMS-D8 was crossed with the recurrent parent [*N (rf*_*2*_*rf*_*2*_*)*], which has excellent agronomic characteristics. Beginning in the BC_1_F_1_ generation, the codominant InDel marker 1327 was used to track the restorer gene in each generation, and the other markers were used for further verification. Only those individuals verified by the markers were chosen as the female parent for successive backcrosses. In the BC_4_F_1_ population, 120 individuals were randomly selected, and then InDel 1327 was used to perform segregation analysis. The individuals verified by the markers as homozygous at the restorer gene locus were test-crossed with the sterile line [*S (rf*_*2*_*rf*_*2*_*)*] to determine the segregation of the fertility phenotype in the offspring under field conditions. The *atpA* SCAR marker distinguishes the CMS cytoplasm from other types of cytoplasm [2]*.* Here, the InDel 1327 marker was combined with the *atpA* SCAR marker to identify hybrids in the CMS-D8 system.

### Real-time quantitative PCR (qRT-PCR) analysis

The annotated genes in the interval were analysed, and real-time quantitative PCR was performed to identify PPR family genes and differentially expressed genes that were selected based on anther transcriptome data of the CMS-D8 system (unpublished data). Total RNA was isolated using the Sigma Spectrum Plant Total RNA Kit (Sigma-Aldrich, USA) according to the manufacturer’s protocol. Reverse transcription was conducted using the PrimeScript™ RT Reagent Kit (TaKaRa, Beijing, China). Trans Start® Top Green qPCR Super Mix (Trans gen, Beijing, China) was used according to the manufacturer’s instructions to conduct qRT-PCR of the genes. The internal control gene used for qRT-PCR was the cotton *His3* gene (i.e., *histone 3*) with primers of *GhHIS3F* and *GhHIS3R*, and relative gene expression levels were calculated using the 2^-△△CT^ method [89], as described in detail in previous studies [90–92]. Each gene in each sample was analyzed with three replicates and two technical replicates. All primers are listed in Table S1.

## Supplementary Information

**Additional file 1: Table S1** The primers of qRT-PCR. **Table S2** Genotype of SNP site. **Table S3** The primers of InDel markers. **Table S4** The FPKM of 67 genes in CMS-D8 system.

**Additional file 2: Fig. S1** The full gel of InDel1599, InDel6989 and InDel1327, *A* sterile line, *B* maintainer line. *R* restorer line

**Additional file 3: Fig. S2** The full gel of InDel8956 and InDel5699, *A* sterile line, *B* maintainer line, *R* restorer line.

**Additional file 4: Fig. S3** BC_4_F_1_ plants were screened with InDel 1327, *M* marker, *H Rf*_*2*_ heterozygous plants, *C* plants lacking the restorer gene *Rf*_*2*_.

**Additional file 5: Fig. S4** The full gel of InDel1327, *A* sterile line, *B* maintainer line, *R* restorer line, *F*_*1*_ A line ×R line.

**Additional file 6: Fig. S5** The full gel of *atpA SCAR, A* sterile line, *B* maintainer line, *R* restorer line, *F*_*1*_ A line ×R line.

## Data Availability

The datasets generated and analysed during the current study are available in the NCBI Sequence Read Archive (SRA) database under Bioproject PRJNA685585 repository (https://www.ncbi.nlm.nih.gov/bioproject/PRJNA685585).

## Abbreviations

BSA: Bulked segregant analysis
CMS: Cytoplasmic male sterility
MAS: Marker assisted selection
PPR: Pentapeptide repeat
*Rf*: Restorer of fertility
SNP: Single nucleotide polymorphisms
InDel: Insertions/deletions
