# Supplementary material for: Physical mapping and InDel marker development for the restorer gene Rf2 in cytoplasmic male sterile CMS-D8 cotton
Source: BMC Genomics. 2021 Jan 6;22:24. doi: 10.1186/s12864-020-07342-y (PMC7789476; doi:10.1186/s12864-020-07342-y)
Supplement: Supplementary file 1 — Additional file 1: Table S1 The primers of qRT-PCR. Table S2 Genotype of SNP site. Table S3 The primers of InDel markers. Table S4 The FPKM of 67 genes in CMS-D8 system. [file 12864_2020_7342_MOESM1_ESM.docx]

Table S1 The primer of qRt-PCR

| Primer | Sequence（5’-3’） |
| --- | --- |
| *GhHIS3F* | CGGTGGTGTGAAGAAGCCTCAT |
| *GhHIS3R* | AATTTCACGAACAAGCCTCTGGAA |
| *Gh_D05G3356F* | GCTGAAGGGGTTGTGTAAGACCG |
| *Gh_D05G3356R* | CAAAGGCAGTCAATGACGGTGC |
| *Gh_D05G3357F* | GAAGCATACAGGTTATTTGGGAG |
| *Gh_D05G3357R* | AAGATCCATAAATAAGGTGGCAG |
| *Gh_D05G3359F* | TGAGAAGGGTTGTGCACCTAA |
| *Gh_D05G3359R* | CTCTCGACCTCGTGCACTATT |
| *Gh_D05G3378F* | AGGTTGTTTGGGAGCATGGG |
| *Gh_D05G3378R* | GTTGGAAAGGAACCCCCGAA |
| *Gh_D05G3380F* | ATTGGGTGTTTCCCACGATGTTT |
| *Gh_D05G3380R* | AGGCTCAACACCTAACTTCAGCA |
| *Gh_D05G3389F* | TGTTGGCAGCTGAAAATTGGCG |
| *Gh_D05G3389R* | AGGCATTCGATGTGGGTAGCAA |
| *Gh_D05G3391F* | AGGGGATGATGGCTTCGAGA |
| *Gh_D05G3391R* | CTTGCAATGCGCATGAACCA |
| *Gh_D05G3392F* | GGGTTTTGTAAAGCTGGGCA |
| *Gh_D05G3392R* | GCAAAGGAACCCCCGAATCA |
| *Gh_A05G3374F* | ATAGCATGCGGCACCAGTTTGA |
| *Gh_A05G3374R* | GGCGACACGTAAGACATCACCA |
| *Gh_A05G3407F* | TTGGCTGCACTTTTTGGCATGG |
| *Gh_A05G3407R* | GGGGCTGCGATGGAAAGATTCA |
| *Gh_A05G3417F* | TGGACAAATAGTCCGAGCAGAGC |
| *Gh_A05G3417R* | GTCCTCAACTGGATACCCACGA |

Table S2 Genotype of SNP site

| SNP ID | Chr | Pos | Ref | Alt |
| --- | --- | --- | --- | --- |
| 650 | D05 | 25905681 | T | C |
| 32097 | D05 | 28011614 | G | C |
| 69121 | D05 | 30035059 | A | T |
| 109174 | D05 | 31910905 | A | C |
| 151819 | D05 | 33976324 | C | G |
| 201885 | D05 | 36074162 | C | T |
| 240696 | D05 | 37826288 | A | G |
| 276756 | D05 | 39810282 | A | G |
| 315671 | D05 | 42079598 | C | A |
| 366673 | D05 | 44101878 | T | C |
| 414992 | D05 | 46111860 | C | T |
| 449971 | D05 | 47997978 | G | A |
| 491977 | D05 | 49817710 | A | G |
| 510719 | D05 | 51034502 | A | G |
| 540975 | D05 | 52440564 | T | A |
| 543259 | D05 | 52548967 | T | C |
| 549740 | D05 | 53105753 | A | C |
| 563981 | D05 | 54110144 | C | A |
| 583963 | D05 | 55082422 | A | T |
| 597385 | D05 | 55994316 | T | A |
| 615526 | D05 | 57015230 | G | C |
| 636309 | D05 | 58028000 | C | G |
| 655668 | D05 | 59027039 | T | A |
| 664754 | D05 | 59760961 | G | T |

Table S3 The primer of InDel markers

| Primer | Sequence（5’-3’） |
| --- | --- |
| InDel3467F21 | TCACCCTTGGTTTTCAAGGTC |
| InDel3467R20 | GCCAGCCATTTACCCTATGC |
| InDel9824F21 | TCAGAGCAGCATTGTGACGTG |
| InDel9824R21 | ATGGGATATGACCCTCCAAGG |
| InDel3095F25 | ACCTAGAGAATACAATTCCAGATTT |
| InDel3095R21 | ATTGGAAATGGCAGGGTTCAA |
| InDel8956F22 | GCCACTAAACTCAACTTTCTAAT |
| InDel8956R24 | GTAGGAGAGAGGTAAAAAAAAAAG |
| InDel4248F21 | ACCGACATTGTAACACCCTGA |
| InDel4248R20 | CCGCCAAGGAATAGGCTAGG |
| InDel7014F23 | ACACAAGTGGTTCCATATCAGCA |
| InDel7014R20 | TTTGTGCACTTATGCGCCAG |
| InDel0425F20 | TGAACTTCCGTGTGAGCGAA |
| InDel0425R20 | AGCCGAATGAAGAACCCGAA |
| InDel5699F20 | TGGAAACCACTCGAACCGAA |
| InDel5699R20 | AACCGATGCAACCGACTCAA |
| InDel1599F20 | GCACGGGGTGACTTGAAATG |
| InDel1599R20 | GGTATTGAATTCCGGGGTGC |
| InDel6989F23 | TTTTTCAGCAAAGTTTAAGGGCT |
| InDel6989R20 | TCAGTGGTTGAGGCTTTCCC |
| InDel5936F23 | CAACGTTGTCACTTATTCCAAGC |
| InDel5936R20 | GGCACATGATGAGGCACTCT |
| InDel1327F21 | AATCCCACCTCTCCTCTCCAA |
| InDel1327R21 | TCTATGACGGCAATCGCAACA |
| InDel1118F23 | ATTGTACAACCCAATTTTGCCCA |
| InDel1118R19 | GGCGGCTGAAATCTAGGGG |
| InDel9342F22 | CATGGCCGACATTGAAAAGAGA |
| InDel9342R20 | GCGCCTTTTGCATCAGAGTG |
| InDel6325F21 | TCCTTCGCATTGGTTTCTGAG |
| InDel6325R21 | AACCAATGACCCAATTCTCCA |
| InDel7191F20 | GGTCTCTCCCCTTGGTTTGG |
| InDel7191R20 | TGGGGCACCATTCCCATAAG |

Table S4 The FPKM of 67 genes in CMS-D8 system

| Gen ID | R_mean_fpkm | A_mean_fpkm | B_mean_fpkm |
| --- | --- | --- | --- |
| *Gh_D05G3355* | 0 | 0.09 | 0 |
| *Gh_D05G3356* | 1.57 | 1.73 | 1.54 |
| *Gh_D05G3357* | 2.45 | 8.77 | 7.77 |
| *Gh_D05G3358* | 0.28 | 8.51 | 7.71 |
| *Gh_D05G3359* | 0.61 | 5.48 | 5.15 |
| *Gh_D05G3360* | 0.35 | 2.67 | 2.35 |
| *Gh_D05G3361* | 4.42 | 7.29 | 9.33 |
| *Gh_D05G3362* | 1.68 | 4.28 | 4.52 |
| *Gh_D05G3363* | 9.72 | 29.88 | 42.37 |
| *Gh_D05G3364* | 0.01 | 0.07 | 0.16 |
| *Gh_D05G3365* | 0.01 | 0 | 0 |
| *Gh_D05G3367* | 0.01 | 0 | 0.01 |
| *Gh_D05G3368* | 0.32 | 0.18 | 0.28 |
| *Gh_D05G3369* | 0.06 | 0.04 | 0.02 |
| *Gh_D05G3370* | 0.01 | 0 | 0 |
| *Gh_D05G3371* | 0 | 0 | 0 |
| *Gh_D05G3372* | 0 | 0 | 0 |
| *Gh_D05G3373* | 0 | 0.01 | 0.05 |
| *Gh_D05G3374* | 0.46 | 0.02 | 0.01 |
| *Gh_D05G3375* | 0.04 | 0.01 | 0 |
| *Gh_D05G3376* | 0.11 | 0.01 | 0.01 |
| *Gh_D05G3377* | 0.72 | 1.57 | 1.53 |
| *Gh_D05G3378* | 1.53 | 3.35 | 3 |
| *Gh_D05G3379* | 0 | 0.23 | 0.26 |
| *Gh_D05G3380* | 2.71 | 4.57 | 4.08 |
| *Gh_D05G3381* | 0.21 | 0.19 | 0.07 |
| *Gh_D05G3382* | 0.14 | 0.54 | 0.45 |
| *Gh_D05G3385* | 0.39 | 1.38 | 1.06 |
| *Gh_D05G3386* | 1.44 | 13.36 | 11.41 |
| *Gh_D05G3387* | 0.02 | 0.04 | 0.03 |
| *Gh_D05G3388* | 0.29 | 0.03 | 0.04 |
| *Gh_D05G3389* | 1.73 | 1.87 | 1.67 |
| *Gh_D05G3390* | 0 | 0 | 0 |
| *Gh_D05G3391* | 0.15 | 3.75 | 3.7 |
| *Gh_D05G3392* | 0.31 | 0.98 | 0.97 |
| *Gh_D05G3393* | 1.76 | 7.36 | 5.1 |
| *Gh_D05G3394* | 2.58 | 6.07 | 5.01 |
| *Gh_D05G3395* | 0 | 0.03 | 0 |
| *Gh_D05G3398* | 0.02 | 0 | 0 |
| *Gh_D05G3399* | 0.02 | 0.03 | 0.06 |
| *Gh_D05G3400* | 0.03 | 0 | 0 |
| *Gh_D05G3402* | 0.01 | 0.23 | 0.14 |
| *Gh_D05G3403* | 0.04 | 0.04 | 0.02 |
| *Gh_D05G3404* | 0 | 0.06 | 0.05 |
| *Gh_D05G3405* | 0.01 | 0.31 | 0.13 |
| *Gh_D05G3406* | 0.32 | 3.05 | 1.69 |
| *Gh_D05G3407* | 1.83 | 0.3 | 0.36 |
| *Gh_D05G3412* | 0.04 | 0.41 | 0.37 |
| *Gh_D05G3413* | 0 | 0 | 0.01 |
| *Gh_D05G3414* | 0.03 | 0.24 | 0.29 |
| *Gh_D05G3415* | 0.01 | 0.18 | 0.21 |
| *Gh_D05G3416* | 0.07 | 1.07 | 0.86 |
| *Gh_D05G3417* | 0.56 | 0.06 | 0.06 |
| *Gh_D05G3418* | 0.01 | 0 | 0.01 |
| *Gh_D05G3419* | 0.01 | 0.01 | 0 |
| *Gh_D05G3420* | 0.05 | 0.02 | 0 |
| *Gh_D05G3423* | 0 | 0.49 | 0.4 |
| *Gh_D05G3424* | 0.06 | 0.04 | 0 |
| *Gh_D05G3425* | 0.03 | 0.01 | 0 |
| *Gh_D05G3429* | 0.57 | 4.82 | 4.45 |
| *Gh_D05G3430* | 5.13 | 4.13 | 4.1 |
| *Gh_D05G3431* | 1.53 | 12.25 | 12.55 |
