# Supplementary figures and images for: Physical mapping and InDel marker development for the restorer gene Rf2 in cytoplasmic male sterile CMS-D8 cotton
Source: BMC Genomics. 2021 Jan 6;22:24. doi: 10.1186/s12864-020-07342-y (PMC7789476; doi:10.1186/s12864-020-07342-y)

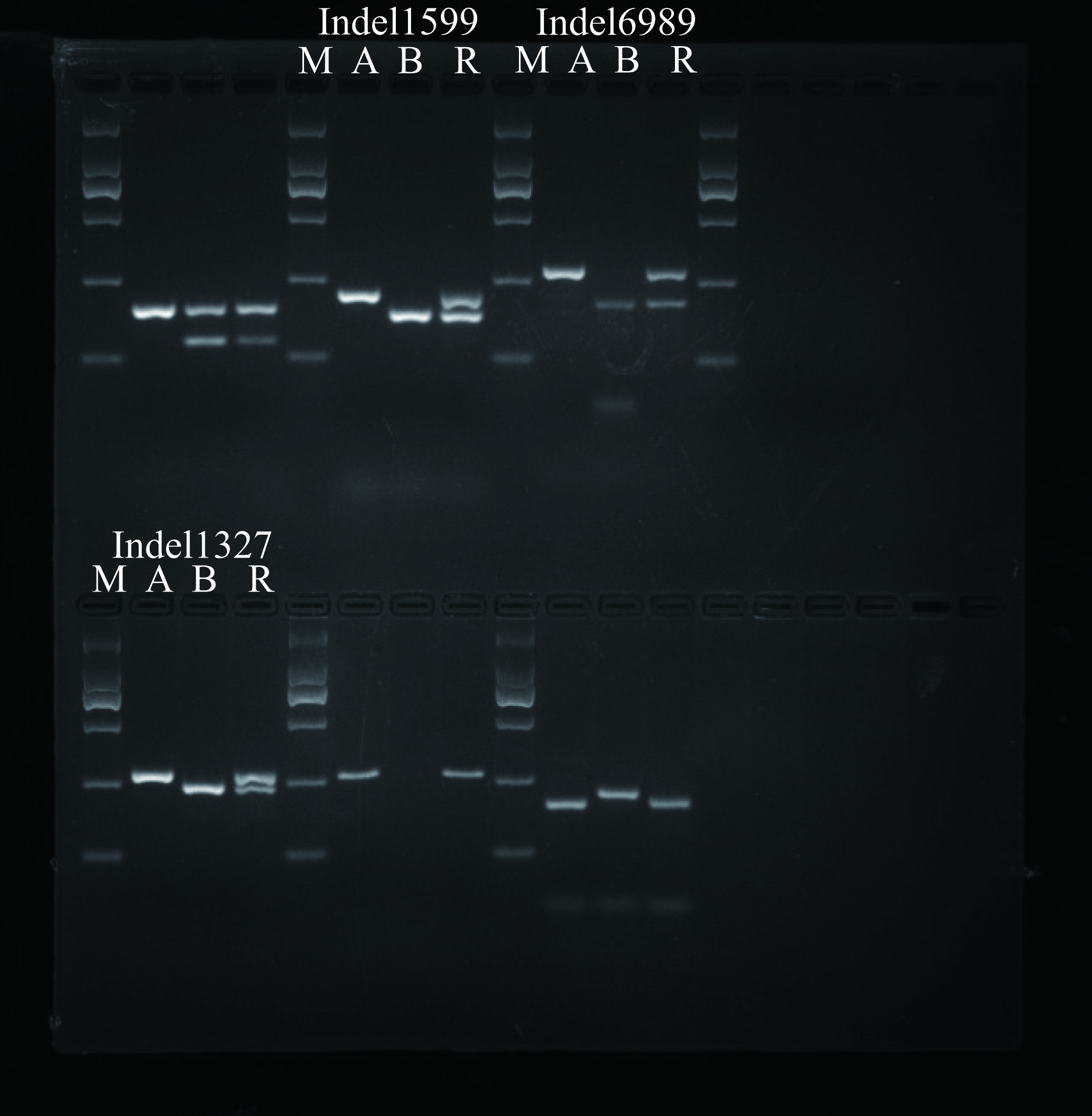

Supplement: Supplementary file 2 — Additional file 2: Fig. S1 The full gel of InDel1599, InDel6989 and InDel1327, A sterile line, B maintainer line. R restorer line [file 12864_2020_7342_MOESM2_ESM.tif]

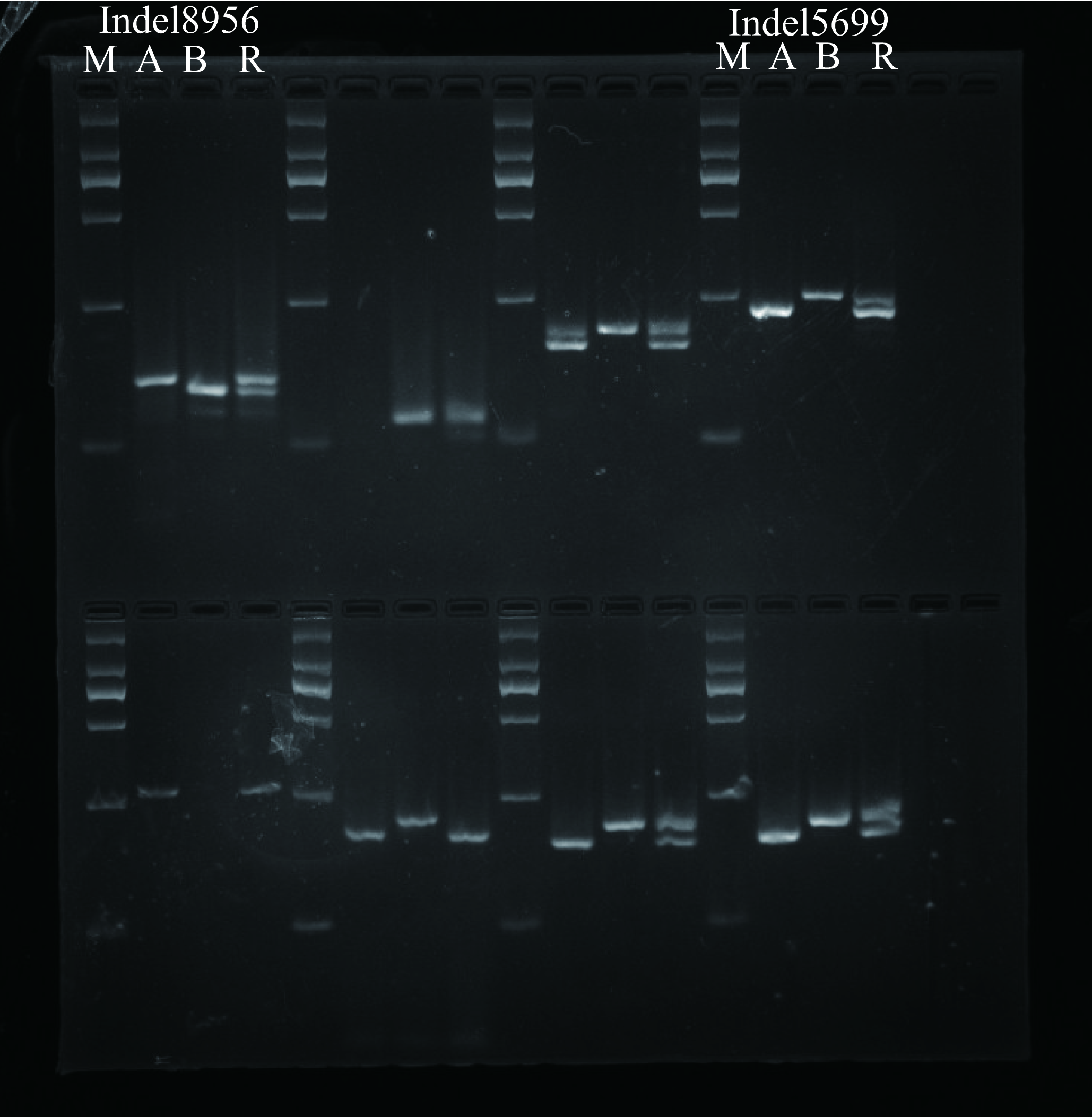

Supplement: Supplementary file 3 — Additional file 3: Fig. S2 The full gel of InDel8956 and InDel5699, A sterile line, B maintainer line, R restorer line. [file 12864_2020_7342_MOESM3_ESM.tif]

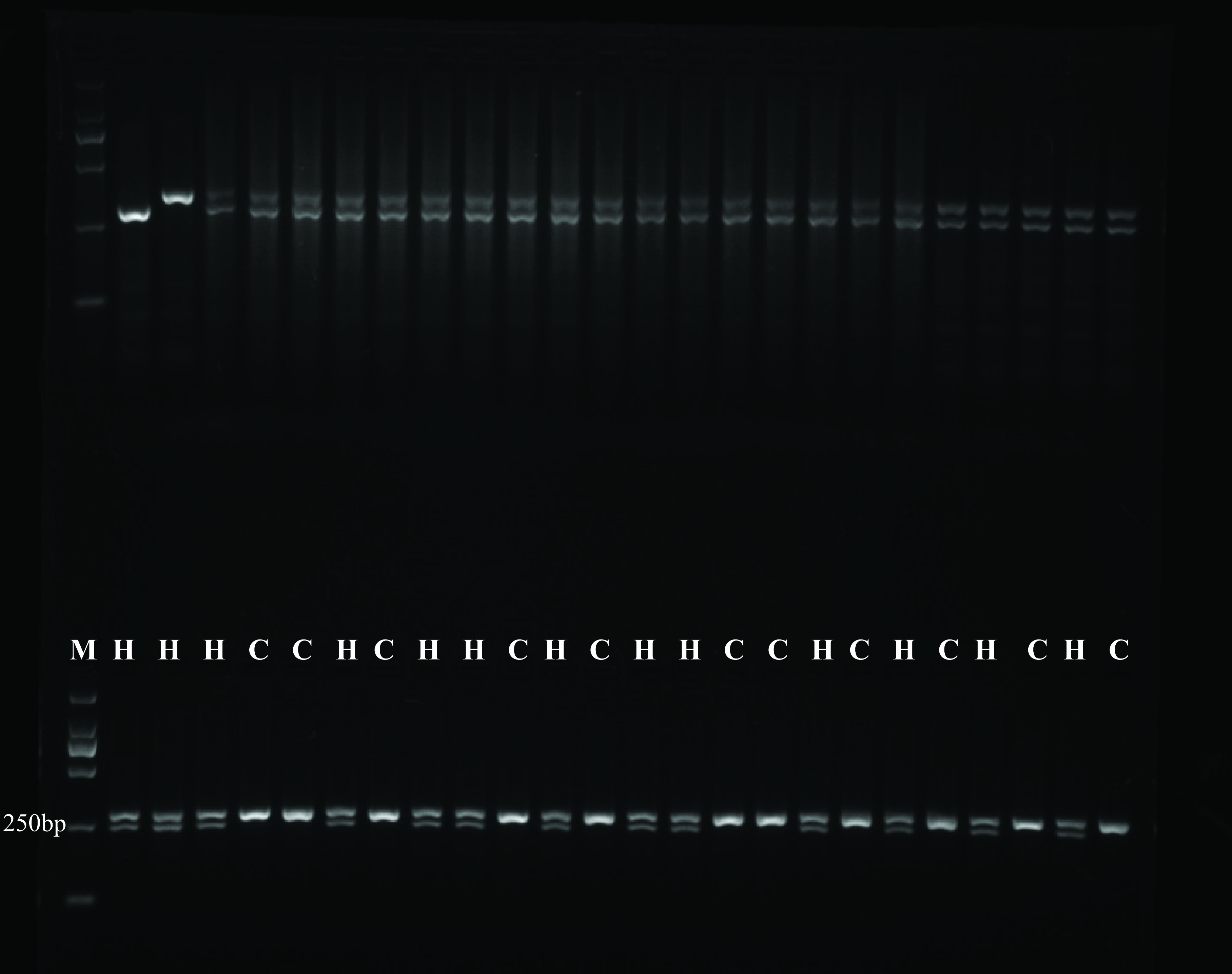

Supplement: Supplementary file 4 — Additional file 4: Fig. S3 BC4F1 plants were screened with InDel 1327, M marker, H Rf2 heterozygous plants, C plants lacking the restorer gene Rf2. [file 12864_2020_7342_MOESM4_ESM.tif]

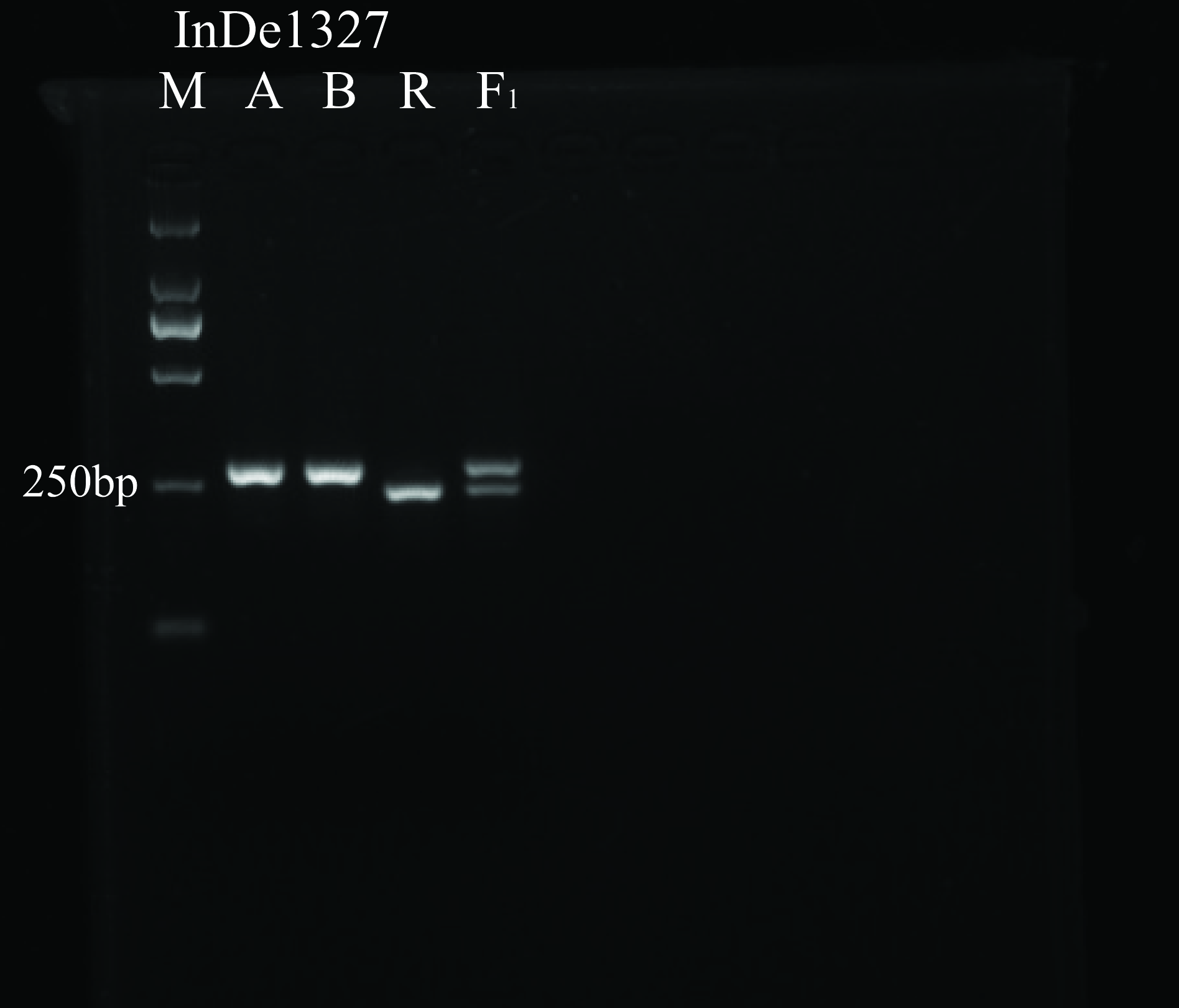

Supplement: Supplementary file 5 — Additional file 5: Fig. S4 The full gel of InDel1327, A sterile line, B maintainer line, R restorer line, F1 A line ×R line. [file 12864_2020_7342_MOESM5_ESM.tif]

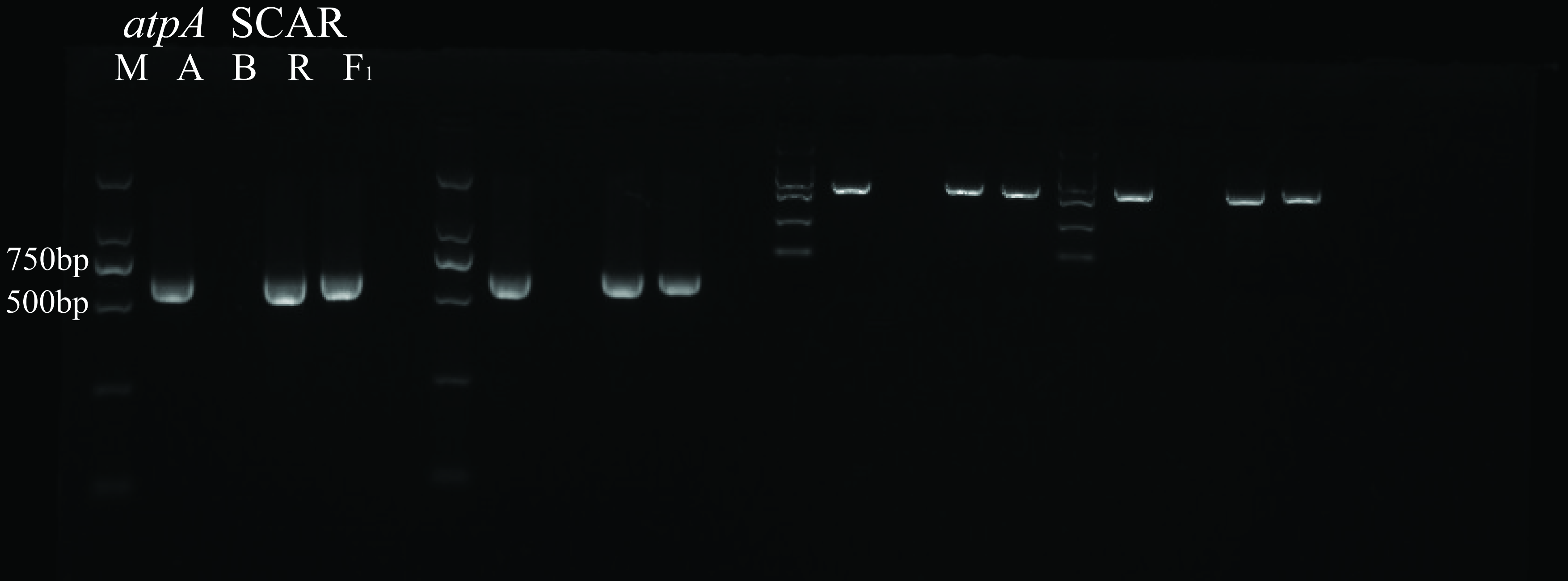

Supplement: Supplementary file 6 — Additional file 6: Fig. S5 The full gel of atpA SCAR, A sterile line, B maintainer line, R restorer line, F1 A line ×R line. [file 12864_2020_7342_MOESM6_ESM.tif]
